# Comprehensive Detection of Genes Causing a Phenotype Using Phenotype Sequencing and Pathway Analysis

**DOI:** 10.1371/journal.pone.0088072

**Published:** 2014-02-26

**Authors:** Marc Harper, Luisa Gronenberg, James Liao, Christopher Lee

**Affiliations:** 1 Institute for Genomics and Proteomics, University of California Los Angeles, Los Angeles, California, United States of America; 2 Dept. of Chemistry & Biochemistry, University of California Los Angeles, Los Angeles, California, United States of America; 3 Dept. of Computer Science, University of California Los Angeles, Los Angeles, California, United States of America; 4 Molecular Biology Institute, University of California Los Angeles, Los Angeles, California, United States of America; 5 Department of Chemical and Biomolecular Engineering, University of California Los Angeles, Los Angeles, California, United States of America; INRA Clermont-Ferrand Research Center, France

## Abstract

Discovering all the genetic causes of a phenotype is an important goal in functional genomics. We combine an experimental design for detecting independent genetic causes of a phenotype with a high-throughput sequencing analysis that maximizes sensitivity for comprehensively identifying them. Testing this approach on a set of 24 mutant strains generated for a metabolic phenotype with many known genetic causes, we show that this pathway-based phenotype sequencing analysis greatly improves sensitivity of detection compared with previous methods, and reveals a wide range of pathways that can cause this phenotype. We demonstrate our approach on a metabolic re-engineering phenotype, the PEP/OAA metabolic node in *E. coli*, which is crucial to a substantial number of metabolic pathways and under renewed interest for biofuel research. Out of 2157 mutations in these strains, pathway-phenoseq discriminated just five gene groups (12 genes) as statistically significant causes of the phenotype. Experimentally, these five gene groups, and the next two high-scoring pathway-phenoseq groups, either have a clear connection to the PEP metabolite level or offer an alternative path of producing oxaloacetate (OAA), and thus clearly explain the phenotype. These high-scoring gene groups also show strong evidence of positive selection pressure, compared with strictly neutral selection in the rest of the genome.

## Introduction

Discovering what genes cause a specific phenotype poses several experimental and analytical challenges, and there are several approaches in the literature for causal gene identification including direct identification of causal mutations from naturally evolving populations growing in the prescence of isobutanol [1] [2], using transposon insertions to detect antibiotic targets [3], use of chemical mutagenesis to produce randomly generated mutants and subsequent high-throughput sequencing to identify key mutation [4] [5] [6]. In particular, the method described in [6], called phenotype sequencing, combines the last approach with sequencing techniques to produce more information at a substantially reduced total cost. The results of the first phenotype sequencing experiment were further verified in the study by Minty et al [1], which found specific causal mutations in many of the genes identified by phenotype sequencing (and also verified partially by [2]; see also [7]). See [8] and [9] for more on pooling methods.

**Figure 1:**
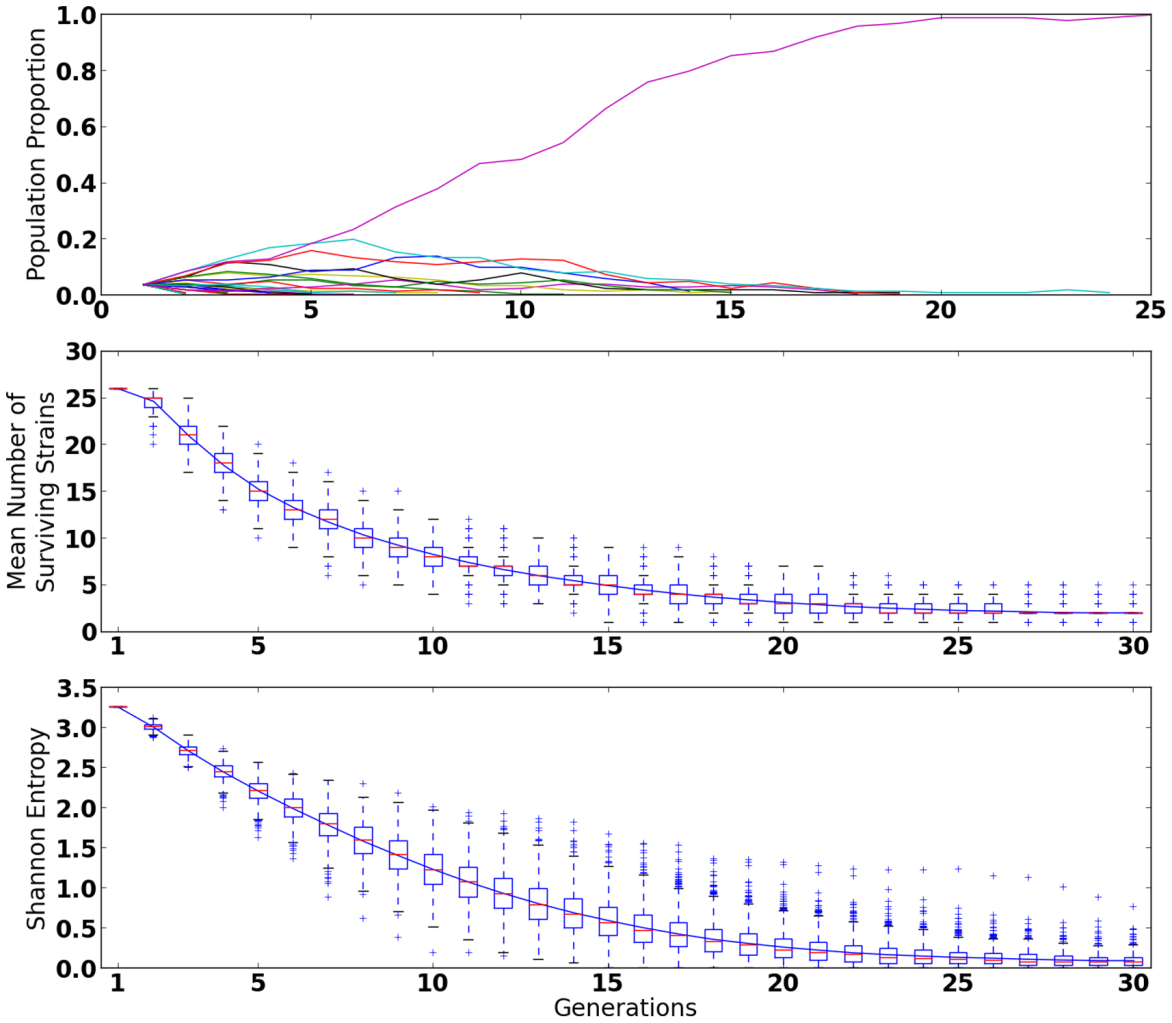
1000 Simulations of the Wright-Fisher Selective Dynamics [58] of a Randomly Mutagenized Population. A. (Top) a simulation of 26 strains of various fitnesses that grow exponentially from a founder population of individual mutants to a carrying capacity under Wright-Fisher selection dynamics. The results of a single simulation show that one mutant dominates the population after a small number of generations. Note diversity is lost due not only to selection, but also genetic drift. B. (Middle) As reproduction and selection proceeds, the mean number of distinct strains decreases very quickly. On average half of the strains are lost after just 6–7 generations. C. (Bottom) Similarly, the mean Shannon entropy [59] of the population distribution also decreases quickly. This differs from (B) in that the population proportions are also taken into account.

Many such methods, while successful, have substantial drawbacks in terms of efficiency and comprehensivity of detection, total labor required to create mutants and verify mutations as causal, and overall cost. Unless the mutagenesis density is very low, there can be many mutations that must be checked; if there is only a single mutation in each mutant, causes of complex phenotypes requiring more than one mutation may be missed. Naturally evolved strains typically both have fewer mutations (10–20 typically) and a larger fraction of these directly contribute to the phenotype [10] [11] [12] [13] [14] [15], with in some cases as few as 3 mutations per strain [16] [17] or more than 40 [18]. On the other hand, natural evolution typically involves a mixture of many mutants competing with each other. Even small differences in selective advantage will tend to give a winner-take-all outcome, in which the ''top'' mutant takes over the culture, and other causes of the phenotype are obscured. This can occur even over a relatively short period of competitive culture (illustrated in Fig. 1). Hence if mutants are allowed to compete, detection of smaller contributors to the phenotype can be washed out by the growth of other mutants. This means that only some of the causes of a particular phenotype will be detected. In particular, if there is a ''trivial'' way to get the phenotype, this can obscure the interesting, non-obvious causes of the phenotype.

While mutagenesis can aid the production of mutants with the desired phenotype, it also elevates the total number of mutations in each strain (often 50 – 100 mutations [19] [20], of which perhaps only one actually causes the phenotype). Dissecting these many candidate mutations experimentally can be laborious, so we employ statistical methods to detect which mutations are most likely to be causal.

Given these challenges, it would be very useful to have reliable high-throughput methods for comprehensively identifying all the genetic causes of a phenotype. Three features seem crucial for this goal. First, sufficient mutagenesis coverage is required to hit all the potential causes of the phenotype. Note that this may require mutating two or more genes simultaneously to achieve the desired phenotype. For a gene to be identified with any kind of statistical significance in a high-throughput (genome-wide) analysis, the ''target'' set of mutations in a gene that can actually cause the phenotype must be hit not just once but multiple times in independent strains. Second, the different mutant strains (representing independent mutagenesis events) must be screened non-competitively, e.g. by either picking only one colony from each independent experiment, or by forgoing long growth rescue in liquid medium to avoid multiple colonies arising from genetically identical daughter cells of a single mutant. This ensures that the different strains with the phenotype will be independent mutation events that represent an unbiased sampling of the diverse possible causes of the phenotype. High-throughput sequencing of the independent mutant strains, yielding the total number of times a gene is independently ''hit'' by mutations across all the strains, can then directly reveal genes that cause the phenotype [6]. We refer to this bioinformatic approach as ''phenotype sequencing''. Third, to attain sensitive and *comprehensive* discovery of the causal genes, the analysis must be able to combine signals across multiple genes that function together, e.g. in the same pathway. When multiple genes in a pathway can cause the same phenotype, this ''splits'' the signal (concretely, the number of observed mutations) among them, making it much harder to detect. For example, our first phenotype sequencing analysis did not obtain a statistically significant score for some genes that are known to cause the phenotype, even though they were relatively highly ranked (due to having more mutations than expected by random chance) [6]. Combining signals from multiple such genes in a pathway could greatly improve sensitivity and hence allow for comprehensive discovery.

**Figure 2:**
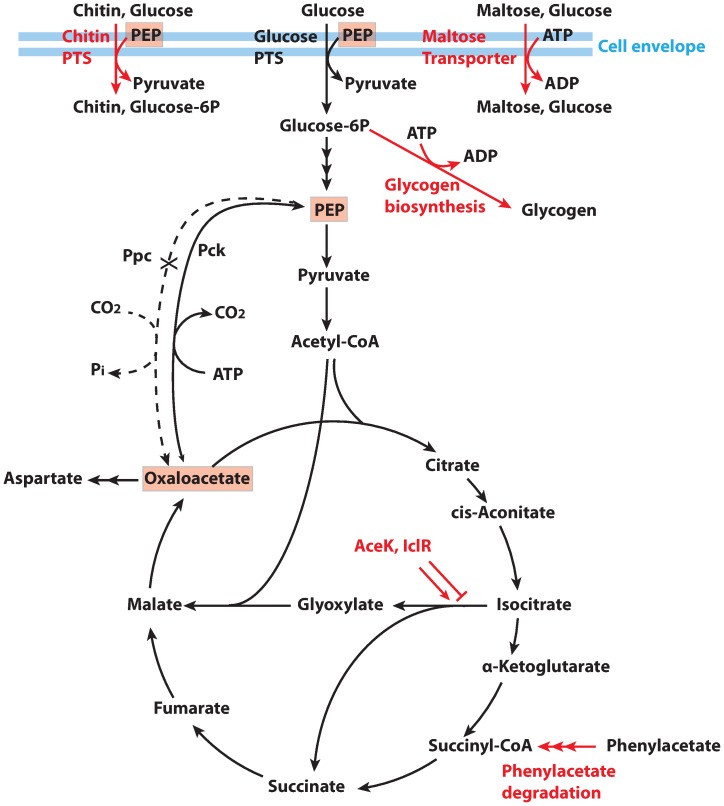
Schematic of metabolic pathways affected by Ppc knockout (dotted line). The 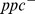 strain requires additional oxaloacetate to grow. Growth is achieved through direct synthesis of oxaloacetate by alternative pathways such as the glyoxylate shunt or pck, or through an increase of PEP levels, which drives flux through these pathways. The top seven mutated pathways identified by pathway phenotype sequencing are shown in red. It has been shown that Ppc knockouts cause increased flux through the glyoxylate shunt [60], consistent with our observed mutations in AceK and IclR. Mutations in PtsI have previously been observed in response to a growth-based selection for increased succinate production, in a scenario where Pck overexpression was also observed [27]. Similarly, deletion of ptsH, which also deactivates the PTS system and increases the intracellular PEP pool, has also been shown to increase succinate yields [48].

As an experimental test, we sought a phenotype that involves many pathways. We therefore chose a metabolic phenotype, namely recovery of ability to grow on glucose by *E. coli* lacking the Phosphoenolpyruvate carboxylase (PPC) enzyme. Oxaloacetate (OAA) is an essential metabolite for the first step of the TCA cycle. Normally, consumption of OAA to produce aspartate is replenished by the PPC enzyme (converting phosphoenolpyruvate (PEP) to OAA; see Fig. 2 for a pathway overview). In the absence of PPC, cell growth becomes limited by the cell's inability to produce OAA. Hence, growing a 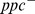 strain on glucose selects for alternative ways of producing OAA.

There is a plethora of experimental data identifying multiple pathways that can contribute to OAA production. Metabolic engineering of the PEP-OAA node has long been studied as a way of modifying the energy balance of the cell [21] [22] [23] [24] [25]. For example, Phosphoenolpyruvate carboxykinase (PEPCK) decarboxylates OAA and activates it to PEP using ATP as a substrate. PEPCK is reversible in some organisms and the reverse PEPCK reaction is more energy efficient than the PPC reaction because it conserves the phosphate from PEP by generating ATP. In *E. coli*, however, PEPCK is not expressed under glucose-grown conditions and therefore the reverse PEPCK reaction cannot substitute for PPC. Recently, with the increased focus on renewable resources from microbes, optimization of the PEP/OAA metabolic node has received renewed interest. Much of the interest has focused on increasing the production of succinate (a high value carboxylic acid of industrial relevance) in *E. coli* and other microbes. In recent studies PPC has been supplemented or replaced with pyruvate carboxylase (PYK) [26], and more often with PEPCK [27], which increases the ATP pool of the cell. This can lead to higher levels of succinate production from a variety of feedstocks [28]. The use of PEPCK and the resulting higher ATP concentration has also been exploited to increase production of malate, OAA [29] or fumarate [30] and even the amount of recombinantly expressed proteins [31]. Overexpressing either a native or heterologous *pepck* gene is one way to compensate for the knockout of *ppc*; this could be one way to rescue an OAA auxotroph strain [27]. However, a number of other pathways affect OAA levels and flux through the PEP node [32].

On the basis of these favorable prospects for involvement of multiple pathways, we mutagenized a parental 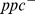 strain, screened for growth on glucose, and analyzed the resulting mutants via phenotype sequencing. Unlike previous reports, we did not eliminate *pflB* [27]. *PflB* encodes a pyruvate-formate lyase, which decarboxylates pyruvate to acetyl-CoA under anaerobic conditions. Since our strain was grown aerobically the pyruvate dehydrogenase complex is responsible for the analogous reaction under these conditions [33]. By not restricting decarboxylation of pyruvate we allowed for mutants that could supply OAA through means other than PEPCK. We mutagenized the 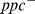 strain and selected for growth on glucose. By performing 24 independent mutagenesis and selection experiments we produced 24 separate mutant strains, identified mutations via pooled sequencing, and identified genetic causes via by pathway-phenoseq and gene-phenoseq.

To assess the possibility of improving detection of multiple pathways, we developed a ''pathway-phenoseq'' analysis that combines mutation signals across each specified pathway. We used pathway information from the EcoCyc database of functionally associated genes in *E. coli* [34]. We also developed bioinformatic validation methods based on gene clustering and independent measures of positive selection. It should be emphasized that pathway-phenoseq seeks to identify which pathways cause a phenotype, but not which individual mutations. So we shall focus our analysis and assessment on its pathway scoring, not on individual mutations.

Note that while causal variants are commonly identified via genome-wide association studies (GWAS), such methods are not appropriate for our dataset because GWAS studies typically rely on thousands of case and control samples, and our samples include one (implicit) control (the parent lacking the phenotype) and 24 independently generated mutants expressing the phenotype. Moreover we exploit the phylogenetic star topology and mutagenesis density resulting from independent mutagenesis experiments, both of which differ greatly from a typical GWAS dataset. Accordingly, statistical and biological assumptions of phenotype sequencing are different than GWAS-based methods such as GSA-SNP [35] and SNP-PRAGE [36], and so these methods, while useful in the appropriate context, are not useful comparisons to the phenotype sequencing statistical model. Instead we will use the widely-known Ka/Ks selection test [37] for comparison.

## Results

### Sequencing of Independent Mutants

Using growth on glucose medium as a selection, 24 mutants with the desired phenotype were produced. The genomic DNA was pooled into 8 libraries each consisting of exactly three strains. These libraries were tagged, combined, and sequenced in a single lane of a high-throughput Illumina Hi-Seq sequencer. The resulting fragments were filtered, aligned to the reference *E. coli* K-12 substr. MG1655 genome sequence, and scanned for sequence variants. Sequencing produced 145 million reads of 100 basepairs each for a total of 14.5 Gb of genomic sequence, of which approximately 118 million reads successfully demultiplexed (had an identifiable tag) and aligned to the reference genome. From the pooled libraries, we identified 2157 SNPS (1450 nonsynonymous, 707 synonymous) after filtering for quality and strand bias (see methods), yielding approximately 100 mutations per strain. These SNPs showed a strong preference for GC-sites in line with the mutagenesis spectrum of NTG [38]. SNPs were detected in 1348 genes; 1012 genes had one or more nonsynonymous mutations.

### Pathway-Phenoseq Analysis

We developed a method for scoring individual pathways, based on the number of non-synonymous mutations occurring in genes in each pathway (see Method for details). As a comprehensive set of *E. coli* pathway annotations, we used the EcoCyc Functionally Associated Groups database, totalling 536 groups [24], of which 336 were hit by non-synonymous mutations in our sequencing dataset. (For simplicity, we will refer to these EcoCyc Functionally Associated Groups as ''pathways''). We applied our scoring method (which we will refer to throughout as ''pathway-phenoseq'') to all 336 pathways, and ranked them by their p-values (Table 1). After the Bonferroni multiple hypothesis correction, the top five pathways (containing 12 genes total) were statistically significant.

**Table 1:** Top 10 gene groups ranked by pathway-phenoseq p-value (Bonferroni corrected for 536 tests).

Group	Genes	p-value (phenoseq)
**PD04099**	*aceK iclR*	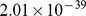
**CPLX0-2101**	*malE malF malG malK lamB*	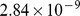
**ABC-16-CPLX**	*malF malE malG malK*	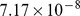
**PD00237**	*malS malT*	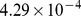
**GLYCOGENSYNTH-PWY**	*glgA glgB glgC*	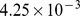
**CPLX-155**	*chbA chbB chbC ptsH ptsI*	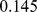
**PWY0-321**	*paaZ paaA paaB paaC paaD paaE paaF paaG paaH paaJ paaK*	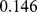
**RNAP54-CPLX**	*rpoA rpoB rpoC rpoN*	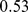
**APORNAP-CPLX**	*rpoA rpoB rpoC*	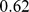
**APORNAP-CPLX**	*rpoA rpoB rpoC rpoD*	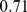

Gene-phenoseq identified three of these genes (*iclR* and *aceK* in pathway PD04099; *malT* in pathway PD00237) as statistically significant (Table 2). Thus pathway-phenoseq detected more than twice as many causal pathways for this phenotype, and four times as many genes as the gene-phenoseq scoring. Even for pathways detected by both, gene-phenoseq had a much weaker p-value (strongest score 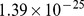) than pathway-phenoseq (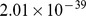); this is expected to be true generally whenever signal is spread over multiple genes in a pathway.

**Table 2:** Top 20 hits ranked by Bonferroni corrected gene-phenoseq p-value computed on non-synonymous SNPs.

Gene	p-value
**iclR**	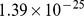
**aceK**	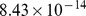
**malT**	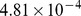
**malE**	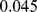
**yjbH**	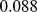
**rplL**	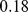
**ydfJ**	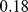
**pgi**	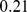
**yhcA**	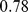
**tyrS**	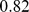
**yjaG**	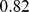
**yeeN**	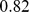
**tig**	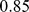
**glgB**	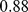
**fdhF**	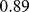
**gntT**	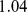
**dbpA**	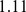
**ydfl**	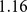
**lysC**	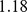
**xylE**	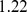

### 0.1 Assessment vs. Experimental Literature

We assessed these results against pathways shown to be involved in this specific phenotype in previous experimental literature. Fong et al. performed natural evolution experiments on a 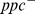 knockout strain selecting for the same phenotype (recovery of the ability to grow on glucose as a carbon source) [24]. After 45 days of growth and selection, they obtained two mutant strains, which had growth rates and glucose consumption rates very similar to the wild type strain, more than double the 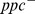 strain on day 0. While Fong et al. did not identify the specific causal mutations, they found that metabolic flux through the glyoxylate shunt (*aceA* and *aceB*) increased, and also that the expression level of these two genes increased. Two other studies found increased flux in the glyoxylate shunt in 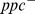 mutants [39], including a rescue of such a mutant by overexpression of the shunt [40].

These data validate our top pathway hit (PD04099, *aceK* and *iclR*), which regulates the glyoxylate shunt [41] [42], the pathway reported by Fong et al. to be specifically up-regulated in association with this phenotype. And a separate set of mutagenesis studies have shown that mutations in *iclR* do indeed increase flux through the glyoxylate shunt [43].

Literature assessment of the top pathways highlights two distinct mechanisms for our growth phenotype (Fig. 2). On the one hand, the glyoxylate shunt provides an alternative source for the cell to make OAA (via the glyoxylate cycle, which produces two OAA molecules for every OAA molecule it consumes). The seventh top hit (PWY0-321) represents the phenylacetate degradation pathway, which produces succinyl-CoA from phenylacetate [44]. This matches the validated glyoxylate shunt mechanism for our growth phenotype; that is, it provides an alternative source for OAA synthesis, from phenylacetate to succinyl-CoA to OAA. On the other hand, the literature indicate that our other pathways instead can increase PEP levels sufficient to induce its conversion to OAA via the PEPCK reverse reaction. For example, the second, third and fourth top hits (CPLX0-2101, ABC-16-CPLX, and PD00237) are all components of the maltose transport pathway, and the sixth pathway (PTS) is a separate transport pathway that consumes PEP to drive transport of glucose. The maltose transporter actively transports glucose into the cell using ATP as energy, whereas other glucose transporters such as PTS consume PEP [45]. So increased maltose transporter activity and decreased PTS activity would both increase PEP levels, and favor its reverse reaction via PEPCK to produce OAA. This has been demonstrated by several experimental studies: a laboratory evolution experiment selecting for increased growth and succinate production identified mutations in PTS that increased flux through PEPCK in the reverse direction [46]. In accordance with Le Chateliers principle, increasing the level of cellular PEP leads to higher reverse PEPCK activity. Zhang et al. also showed that increased expression of the galactose permease (*galP*), in combination with deactivation of the PTS system, increased the PEP efficiency of glucose transport and succinate production [27]. Indeed, a number of studies have reported that mutations in the PEP-dependent phosphotransferase system (PTS) lead to increased flux from PEP to OAA (and on to succinate) [47] [48] [49]. The fifth top hit (GLYCOGENSYNTH-PWY) is not a sugar transport pathway, but instead the glycogen synthesis pathway. While it does not directly consume PEP, it consumes Glucose-6P (G6P), a metabolic precursor of PEP. Loss of (or reduced) function mutations in this pathway would boost G6P and hence PEP levels, as well as glucose and ATP levels, which both decrease the consumption of PEP for glucose transport. It should be emphasized that these previous experimental studies do not prove that mutations in pathways 2–7 can cause our specific phenotype (growth of 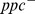 knockout strain on glucose), as they did not test this specific phenotype.

### Bioinformatic Tests

As an additional test of the entire set of top scoring pathways, we computed a p-value for evidence of positive selection (Ka/Ks > 1) within this set (Table 3). Whereas the phenoseq scoring is based on the *total number* of mutations in a region, the Ka/Ks is based on the *ratio* of non-synonymous vs. synonymous mutations (note that the latter are *not* considered by the phenoseq scoring function). The Ka/Ks ratio for the total dataset of 2157 SNPs was 1.0026, consistent with neutral selection, as expected from random mutagenesis. We therefore computed a p-value for the null hypothesis that mutations in the top pathways are drawn from the same background distribution as the total set of mutations (i.e. neutral) using the Fisher Exact Test (see Methods for details).

**Table 3:** Positive Selection evidence for Top 10 gene groups.

Pathway	cumulative p-value	excluding *iclR*, *aceK*, *malT*
**PD04099**	0.0037	N/A
**CPLX0-2101**	0.0044	0.28
**ABC-16-CPLX**	0.0027	0.28
**PD00237**	0.0027	0.29
**GLYCOGENSYNTH-PWY**	0.0020	0.19
**CPLX-155**	0.00043	0.056
**PWY0-321**	0.000068	0.011
**RNAP54-CPLX**	0.000043	0.0063
**APORNAP-CPLX**	0.000043	0.0063
**APORNAP-CPLX**	0.000034	0.0051

The top 10 pathway-phenoseq pathways contained a total of 103 non-synonymous mutations vs. only 21 synonymous mutations, yielding a p-value of 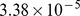. This is strong evidence of positive selection. Even leaving out the genes detected by gene-phenoseq (*iclR*, *aceK*, *malT*), the p-value is 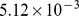. Furthermore, this evidence of positive selection extends throughout the top ten pathways. For example, if one leaves out pathways 6 through 10, the p-value becomes weaker (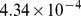, or again leaving out *iclR*, *aceK*, *malT*, 0.056). Indeed the p-value becomes *stronger* (smaller p-value) with each additional pathway added to the analysis, indicating that each pathway shows evidence of positive selection. Note that at the level of single-gene analysis, only one gene (*iclR* with 19 non-synonymous mutations and 1 synonymous mutation) could be detected as showing statistically significant evidence of positive selection (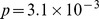); other genes simply did not have enough total mutation counts to attain significance. Only two gene groups (combined into a single meta-gene), *PD04099* containing *iclR* and *aceK* and *TRNA-CHARGING-PWY*, have a Ka/Ks value greater than one with a p-value less than 0.1 from Fisher's exact test. The latter pathway is not obviously connected to the phenotype and is composed of 23 genes involved in many cellular functions.

It is interesting to ask what fraction of the genes in these pathways show evidence of causing the phenotype. It is evident (e.g. from the known experimentally validated genes) that real causal genes are present far below the 0.05 significance threshold of gene-phenoseq scoring (also found to be the case in a previous phenotype sequencing experiment [6].) To assess this, we took the top 50 gene-phenoseq genes, and asked what pathways were strongly enriched (Table 4). Given a top list of genes, one can assess whether they cluster within specific subgroups of a standard functional annotation using the hypergeometric p-value test [49]. This analysis identified statistically significant clustering within three EcoCyc pathways. Furthermore, six of the top ten pathways matched the top 10 pathway-phenoseq pathways. These data indicate that at least 9 of the genes in these pathways contribute causally to the phenotype (since they were individually detected among the top 50 gene-phenoseq hits). Only 28 pathways intersected the top 50 list.

**Table 4:** Top 10 gene groups ranked by hypergeometric p-value (Bonferroni corrected for 28 tests).

Group	Genes	Genes in top 20	p-value (hypergeometric)
**ABC-16-CPLX**	*malF malE malG malK*	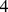	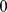
**PD04099**	*aceK iclR*	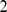	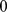
**CPLX0-2101**	*malE malF malG malK lamB*	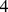	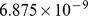
**CPLX-63**	*torY torZ*	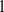	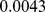
**PD00237**	*malS malT*	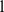	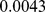
**ABC-42-CPLX**	*alsA alsB alsC*	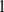	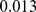
**APORNAP-CPLX**	*rpoA rpoB rpoC*	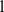	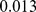
**GLYCOGENSYNTH-PWY**	*glgA glgB glgC*	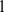	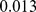
**SECE-G-Y-CPLX**	*secE secG secY*	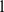	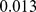
**CPLX0-221**	*rpoA rpoB rpoC fecI*	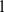	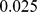

### Causal Mutations Analysis

Finally, we sought to estimate the number of mutations in each group that actually help cause the phenotype (''causal mutations''). In principle, one can estimate this from the observed bias towards non-synonymous mutations (compared with that expected under neutral selection as observed in the total dataset). Specifically, we assume that all causal mutations must be non-synonymous, whereas non-causal mutations are drawn from the background mixture of synonymous + non-synonymous mutations (i.e. neutral selection). We can then estimate the fraction of mutations in each pathway that are causal, since the observed fraction of non-synonymous mutations 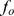 in a pathway will reflect the mix 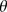 of causal vs. non-causal mutations: 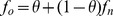



where 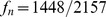 is the fraction of non-synonymous mutations observed in the entire dataset (which almost exactly matches that expected for neutral selection). Then 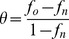



We then estimated the number of causal mutations in a pathway as 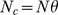, where *N* is the total number of mutations observed in the pathway (Table 5). It is striking, for example, that the estimated number of causal mutations in the top pathway (*iclR* + *aceK*) precisely equals the number of independent mutant strains sequenced (24). This suggests that each strain with this phenotype was mutated once in this pathway, and though there were at least three mutations in this pathway in each pool, we are unable to directly verify a mutation in every strain because of the pooling of mutant strains for sequencing. Nevertheless, given the low amount of mutations per strain, it is statistically unlikely that any particular gene was mutated more than once per strain. The number of causal mutations estimated in the remaining pathways ranged from 4 to 9, suggesting that at least one additional mutation in these other pathways was present in each strain. For each pool of three strains, at least three nonsynonymous mutations were observed in the (*iclR* + *aceK*) pathway, so our data is consistent with the hypothesis that there must be a mutation in this pathway to achieve the phenotype.

**Table 5:** Estimated Causal Mutations in the Top 10 gene groups.

Group	Synonymous Mutations	Non-synonymous Mutations	Causal Mutations
**PD04099**	5	34	24
**CPLX0-2101/ABC-16-CPLX**	6	18	6
**PD00237**	3	11	5
**GLYCOGENSYNTH-PWY**	3	10	4
**CPLX-155**	0	7	7
**PWY0-321**	1	11	9
**RNAP54-CPLX/APORNAP-CPLX/APORNAP-CPLX**	3	12	6

## Discussion

These data indicate that phenotype sequencing can successfully identify genetic causes of a phenotype, directly from high-throughput sequencing data. The top hit from both pathway-phenoseq and gene-phenoseq (glyoxylate shunt) is validated by previous experimental studies of this specific phenotype [39] [24] [40]. Other top pathway hits are also supported by relevant literature [47] [46] [48] [27], but their direct involvement in this specific phenotype has not yet been tested experimentally. We also emphasize that we have not proved *which* of our observed mutations cause the phenotype. The purpose of pathway-phenoseq analysis is not to identify individual causal *mutations*, but rather to identify *pathways* that can cause the phenotype.

Our results suggest that pathway-phenoseq improves sensitivity and comprehensive discovery of the genetic causes of a phenotype, over gene-phenoseq. It detected a statistically significant signal for more than two times as many pathways, and an even greater proportion of genes. Second, our results indicate that independent (non-competitive) mutant strains do indeed reveal a wide variety of genetic causes of a phenotype, in this case: regulators of the glyoxylate shunt; the maltose transport pathway; the glycogen synthesis pathway; and the phosphotransferase system. Third, our analysis suggests that the phenoseq approach is far more sensitive for detecting such ''selection loci'' than standard measures of selection such as Ka/Ks or dn/ds. For example phenoseq detected a single pathway with a p-value of 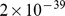 (Bonferroni- corrected), compared with a positive selection p-value on the same pathway of 0.0037 (not even Bonferroni-corrected).

We now consider some further implications and challenges in this work. First, it appears that the number of mutant strains sequenced both in this study (24) and the previous isobutanol tolerance study (32) are inadequate for definitively identifying all genes that contribute to these phenotypes. That is, our results (and other experimental studies) have shown clear evidence for a number of genes causing this phenotype, that failed to attain statistical significance in the gene-phenoseq scoring. In many cases gene-phenoseq scoring ranked these genes highly, but the sample size simply was not large enough to yield a strong p-value. This reflects a fact about our two phenotypes which may apply generally to many other phenotypes: they are complex, and involve many genes, more than can be reliably detected by gene-phenoseq with sequencing of 30 mutant strains. It also illustrates why pathway-phenoseq is needed: in our (admitedly limited) experience, inadequate sensitivity is the key factor limiting discovery.

Second, the same general approach should be applicable to other mutagens and types of mutations. As a minor example, in the current study we did analyze promoter mutations (separately from coding-region mutations), but did not find any significant results (data not shown). The same basic analysis should be applicable to deletion mutations, transposon insert events and any other mutational process for which one can build an adequate neutral model. In the worst case, one could simply obtain data for a control set of strains (i.e. mutated but not screened for the phenotype), to provide an *empirical* model of the mutational bias of the set of genes, that would be used as the null (non-target) model for scoring the results observed after phenotype screening.

Third, it seems interesting to ask how many *causal* mutations are required to produce the phenotype. We have presented a very simplistic way of estimating the number of mutations in each pathway that are actually causal. This already seems to yield intriguing suggestions, for example that the top pathway (glyoxylate shunt regulation) is mutated in essentially *every* strain that has the phenotype, and that this is typically accompanied by a ''second mutation'' in another pathway. It seems likely that more sophisticated approaches to this question will yield useful insights. This is but one example of exploring positive selection signals (concretely, by taking synonymous mutations into account, which phenoseq ignores). Another possible application of positive selection data is suggested by our Table 3: whereas the total mutation dataset is unequivocally neutral (Ka/Ks = 1), the top-ranking pathways show clear evidence of positive selection (Ka/Ks > 1). Thus, it would be interesting to determine (via a robust probabilistic analysis) how far down the list of top-ranked phenoseq pathways this positive selection signal goes (i.e. where does it revert to neutrality). In principle, this could provide a measure of the depth of phenotype selection in the dataset, distinct from the phenoseq p-value.

Finally, we must consider organisms where pathway annotation is lacking (compared with the high level of pathway annotation for *E. coli*). In principle, any source of functional groupings of genes (for example ''Rosetta Stone'', phylogenetic profiles and related non-homology approaches) could be used, in the absence of human-curated pathway annotations. Another interesting possibility is to invert the problem: given a diverse set of easily screenable phenotypes, one could systematically perform phenotype sequencing on many such phenotypes, to obtain observed groupings of genes that appear to ''function together'' in the sense of causing the same phenotype(s). Note that in contrast with a typical ''functional correlation'' analysis (such as on expression levels), even seeing a pair of genes as correlated by a single data point (i.e. both causing one phenotype) would actually be significant. Thus far fewer phenotypes would have to be studied to obtain significant results, than for other functional correlation analyses such as expression levels. Thus phenotype sequencing could itself be used as a high-throughput method for finding functional groupings of genes in less well studied microbial organisms.

## Methods

### Bacterial Strains and Growth Conditions

For strain construction and to prepare samples for NTG mutagenesis strains were grown in standard Luria Bertani medium [50]. Under selective conditions strains were grown in a modified M9 medium (6 g 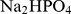, 3 g 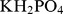, 1 g 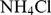, 0.5 g NaCl, 1 mM 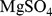, 1 mM 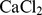, 10 mg vitamin B1 per liter of water) containing 1% glucose.

Mutagenesis was performed on parent strain 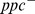. This strain was generated by P1 transduction to delete *ppc* from *E. coli* strain JCL16 (BW25113/F [traD36, proAB+, lacIq ZDM15]) [51], using strain JW3928 from the Keio collection as a P1 donor [52]. This strain is unable to grow on glucose minimal medium.

### NTG Mutagenesis and Selections

Random mutagenesis was performed with -nitro-N-nitrosoguanidine (NTG) as previously described [53]. Briefly, cultures of 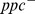 were grown to exponential phase in LB medium, washed twice with 0.1 M citrate buffer and then concentrated two-fold by centrifugation and suspension in 0.1 M citrate buffer (pH 5.5). Samples of 2 mL were exposed to N-nitro-N-nitrosoguanidine (NTG) at a final concentration of 50 mg/mL for 30 minutes at 37C to reach a percentage kill of approximately 50%. The cells were washed twice with 0.1 M phosphate buffer (pH 7.0) and grown in LB for one hour. The cells were then challenged by plating on glucose minimal medium and grown at 37C for 3 days. This procedure was performed on 24 separate samples of 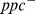, each of which was plated separately to ensure genetically distinct populations of mutants. One colony from each separate NTG experiment was selected, restreaked on selective medium plates to verify the phenotype and then cultured in liquid medium to obtain genomic DNA.

### DNA Library Preparation and Sequencing

Bacterial genomic DNA was prepared from 24 mutant strains using the DNEasy kit from Qiagen using the optional RNAse treatments. The isolated genomic DNA from the mutant strains was pooled in 8 pools, each at a total concentration of 20ng/*µ*L. Equal amounts of DNA from 3 mutant strains were mixed in each of the 8 pools. The pooled samples were then fragmented by sonication to an average size of 100250 bp and confirmed by gel electrophoresis. 8 tagged genomic sequencing libraries (8 different indexes) were constructed using the TruSeq DNA Sample Prep Oligo Kit following the low throughput protocols provided by the manufacturer (Illumina). The final concentration of each of the 8 indexed libraries was measured by QuantiFluor assay and the 8 libraries were mixed in equal proportion at a final concentration of 10 nM. 100bp single end read sequencing was carried out on a single lane of an Illumina Genome Analyzer HiSeq 2000 instrument by the UCLA Broad Stem Cell Research Center High Throughput Sequencing Facility.

### Pathway-Phenoseq Analysis

Short read data were aligned to the reference *E. coli* genome (Genbank accession NC_000913) using Novoalign (Novocraft, Selangor, Malaysia) in single-end mode. Sequence variants were then called using samtools [54] mpileup and bcftools output to VCF format. Only single nucleotide substitutions were found via this analysis, consistent with NTG mutagenesis. We then employed our phenoseq software package to apply a succession of variant filters:

- We excluded variants with inadequate samtools quality scores. Specifically, we required a QUAL value of greater than 90.
- We excluded reported variants with strong evidence of strand bias (i.e. the evidence for the variant came primarily from reads in one direction but not the other). Specifically, we excluded variants with a samtools AF1 p-value of less than 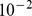. This eliminated a large number of variant calls that appear to have been sequencing errors.
- We excluded variants with samtools allele frequency estimate greater than 50% in any given pool. Concretely, each independent mutant strain is expected to have different mutations, so each mutation should be present in only one out of three of the strains mixed together in one pool.
- We excluded variants that were found in multiple tagged pools. In all cases these were found in all 8 pools, indicating that they were parental strain mutations (i.e. differences versus the reference genome sequence).

We then used the EcoCyc functionally associated gene groups to score pathways as follows:

- we only included non-synonymous mutations in the phenoseq analysis. Specifically, we used the Pygr software package [55] to map the Genbank CDS annotations on the reference genome, to map mutations to CDS (gene) intervals, and to determine their effect on the amino acid translation. Mutations that did not map to a CDS, or did not alter the amino acid translation, were excluded.
- CDS-mapped mutations were mapped to each EcoCyc group using the EcoCyc database.
- The expected mutational cross-section 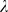 for each EcoCyc group was calculated based on its GC composition, and the total density of all observed mutations on GC sites vs. AT sites over the whole genome.
- We computed a p-value for the null hypothesis that the observed mutations 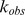 in an EcoCyc pathway were obtained by random chance, under a Poisson model

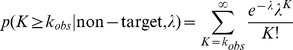



These calculations were performed with the *scipy.stats* module [56].

- We applied a Bonferroni correction to this p-value by multiplying by the total number of EcoCyc pathways groups 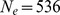.

We performed positive selection tests on these EcoCyc pathway groups as follows:

- for a given set of one or more EcoCyc pathways, we obtained the counts 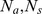 of non-synonymous vs. synonymous mutations in that set of pathways.
- We computed the p-value for obtaining this result under a neutral (i.e. 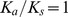), random model:

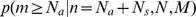



where *N* is the total number of all observed synonymous + non-synonymous mutations in the whole genome, and *M* is the total number of observed non-synonymous mutations in the whole genome. Specifically, we computed this p-value using the one-tailed (''greater'') Fisher Exact Test in R [57].

- Note that since only a single p-value test was performed (on the top-ranked set of pathways), no Bonferroni correction was applied.

Similarly, we computed p-values for pathway ''enrichment'' among the top 50 gene-phenoseq genes using the hypergeometric test, again computed using the Fisher Exact Test in R or scipy [56], with a Bonferroni correction corresponding to the number of pathways that this test was applied to.

All of our code is available under an open source license at https://github.com/cjlee112/phenoseq. Sequence data is available at the NCBI Sequence Read Archive (accession number: SRP018106).
